# Hierarchical Medical System Based on Big Data and Mobile Internet: A New Strategic Choice in Health Care

**DOI:** 10.2196/medinform.6799

**Published:** 2017-08-08

**Authors:** Yaogang Wang, Li Sun, Jie Hou

**Affiliations:** ^1^ Tianjin Medical University Tianjin China

**Keywords:** medical services, continuity of patient care, mobile health

## Abstract

China is setting up a hierarchical medical system to solve the problems of biased resource allocation and high patient flows to large hospitals. The development of big data and mobile Internet technology provides a new perspective for the establishment of hierarchical medical system. This viewpoint discusses the challenges with the hierarchical medical system in China and how big data and mobile Internet can be used to mitigate these challenges.

## Introduction

The hierarchical medical system has become an essential system in many developed countries. It plays an important role of forming the basis for guaranteeing health care [1-4]. The basic working of a hierarchical medical system involves initial diagnoses at primary medical institutions and two-way referrals among hospitals. In China, which has a population of over 1.37 billion [5], there are many problems in the medical system, such as biased resource allocation and extremely high patient flows to large hospitals [6]. Since 2009, China has vigorously promoted the implementation of the hierarchical medical system to realize rational allocation of medical resources, promote the equalization of primary medical services, and reduce the cost of medical services. On the basis of this background, this paper aims to analyze the difficulties of hierarchical medical system in China and opens a dialogue on the challenges associated with the innovation model of the hierarchical medical system by using perspectives based on big data and mobile Internet.

### Difficulties of Hierarchical Medical System in China

**Figure 1:**
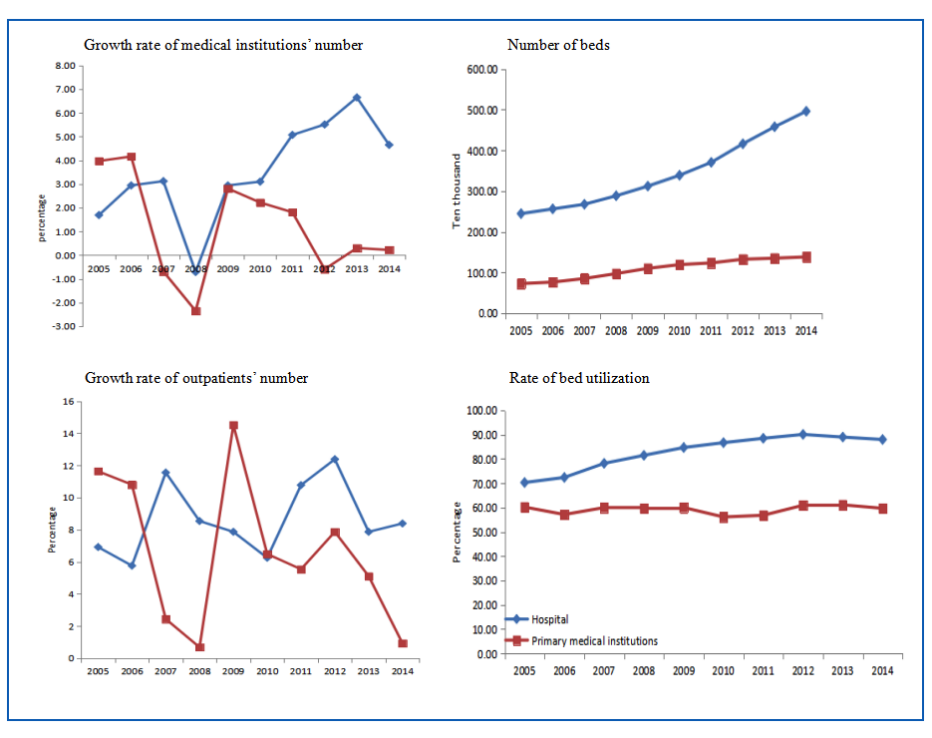
Changes in the numbers of medical institutions, outpatients, beds and bed utilization between hospitals and primary medical institutions from 2005 to 2014 in China.

So far, China has not established an effective model of the hierarchical medical system. In China, hospitals at different levels formed a regional medical consortium (RMC). The government is urging hospitals at different levels in an RMC to strengthen their cooperation and recognize each others’ patient medical results, while encouraging two-way referrals among them. However, an RMC cannot achieve the results that can be expected from a hierarchical medical system. From 2005 to 2014, the number of hospitals in China increased by an average of 716 per year, whereas the primary medical institutions increased by an average of 6785 per year. By contrast, the average annual growth rate of outpatients in hospitals and primary medical institutions were 11.43% and 6.82%, respectively (Figure 1) [7]. The growth rate of outpatients in primary medical institutions has not matched the growth rate of outpatients in institutions. Additionally, the number of beds and the rate of bed utilization increased more in hospitals than in primary medical institutions. Large hospitals are still overcrowded, while primary medical institutions are sparsely populated. High-quality medical resources are concentrated in large hospitals, but primary medical institutions are seriously lacking in medical resources. In addition, the health-information-sharing platforms and associated mechanisms have not been established. Patients’ information cannot be shared among hospitals at different levels so patients cannot enjoy the continuity of medical services between different hospitals. These problems increase the difficulty and cost of medical services in China.

Therefore, the biggest challenge for China is to find a successful way to solve these problems.

### Challenge and Opportunities

In the 21st century, traditional health care has been rapidly changing owing to Internet-based big data and cloud computing [8,9]. Big data is being generated by all digital operations at all times during routine use. Every digital process and social media exchange produces data through systems, sensors, and mobile devices that transmit this data. Therefore, big data is generated by multiple sources with an alarming velocity, volume, and variety. Four continuous stages including the generation, acquisition, storage, and analysis constitute the big data value- chain.

Pervasive Internet access has enabled patients around the world to seek information on the best care available. Additionally, the Internet facilitates efficient communication of medical information globally [10]. Personalized health technologies are also developing rapidly. Sensors, smart-watches, and mobile health apps are strapped to wrists and placed in pockets to monitor and help in modifying health behaviors [11]. Some high-profile devices include Fitbit, Jawbone, Microsoft Band, and Apple smart watch. These technologies help with self-quantification of physical activity and health self-management [12].

In China, “Mobile Internet + Medical Care” oriented by the big data value chain is gradually changing medical practices and processes. The main functions of mobile medical care include making appointments, consulting service, acquiring health information, and providing guidance. By the end of 2016, the number of mobile phone users in China reached over 1.32 billion, while 4G Mobile Internet users accounted for 58.2%(58.2/100) of the total [13]. Meanwhile, the construction of hospital information is developing quickly as the usage of electronic medical records, digital hospitals, telemedicine, and collaborative medical care is on the rise. The development of big data and mobile Internet technology provides a new perspective for the establishment of a hierarchical medical system.

### Hierarchical Medical System Based on Big Data and Mobile Internet

This paper proposes an innovative model of the hierarchical medical system based on big data and mobile Internet that may become a new strategic choice to resolve the imbalance in the availability of medical resources in China.

**Figure 2:**
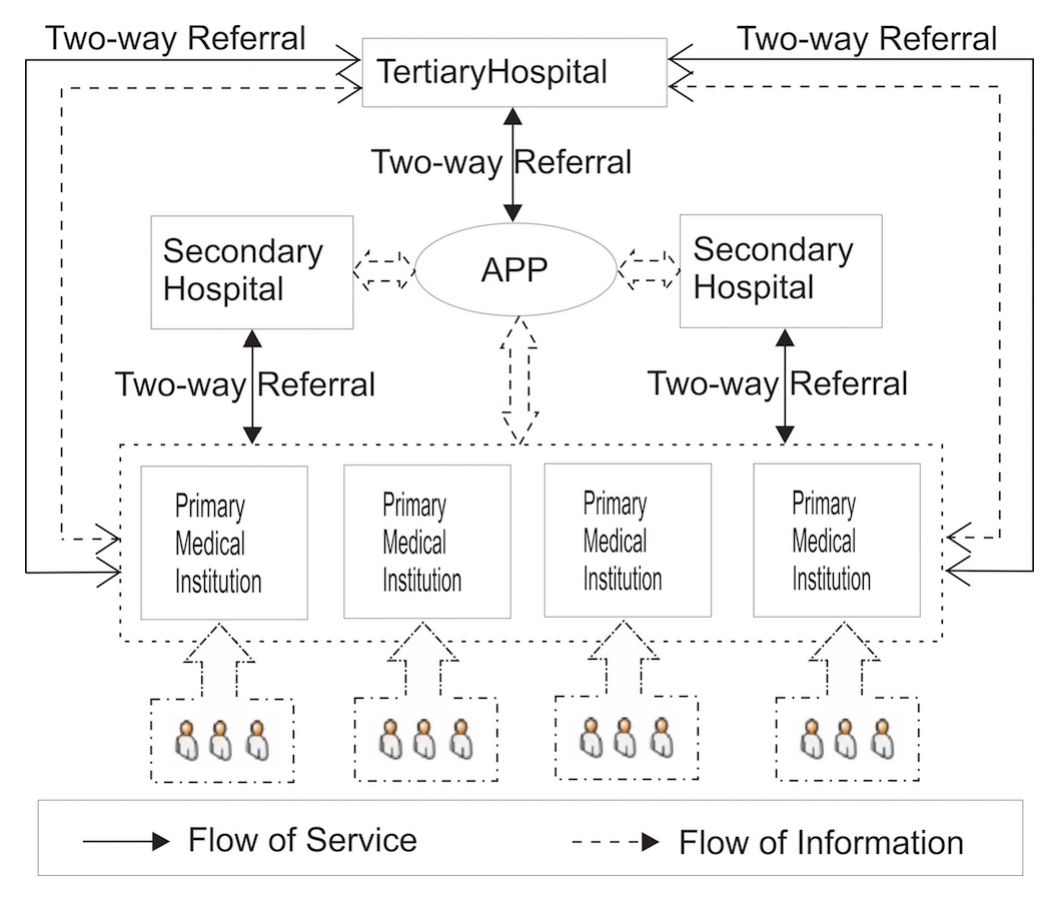
The theoretical model of hierarchical medical system based on big data and mobile Internet.

This innovative model aims to materialize 5 successive medical services. These services would involve linkages at different levels such as institutional, interdisciplinary, interpersonal, patient satisfaction, and management. These linkages have no interruptions by using the Data Sharing and Processing System (DSPS) (Figure 2).

**Figure 3:**
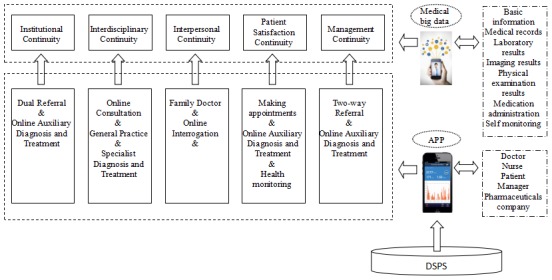
Functions of hierarchical medical system based on big data and mobile Internet.

In this model, medical data can be recorded and shared among hospitals and health facilities at different levels across the DSPS. The important basic patient information including the physical examination file, medical records, laboratory results, imaging results, medication records, self-monitoring, and other relevant information can be transmitted to medical service providers in different institutions, which would result in patients being able to receive continuous medical services when they go to different institutions to receive care. In addition, advanced medical resources in large hospitals can also accessed by the primary medical institutions without the constraints of time and space. Doctors at more advanced hospitals can participate in the medical activities of lower level medical institutions online and help medical professionals in primary medical institutions to improve the quality of their services. Owing to this connectivity and cooperation, service providers at different facilities can become familiar with one another and establish long-term relationships. In addition, patients would have the benefit of receiving continuous medical service at different institutions seamlessly (Figure 3).

**Figure 4:**
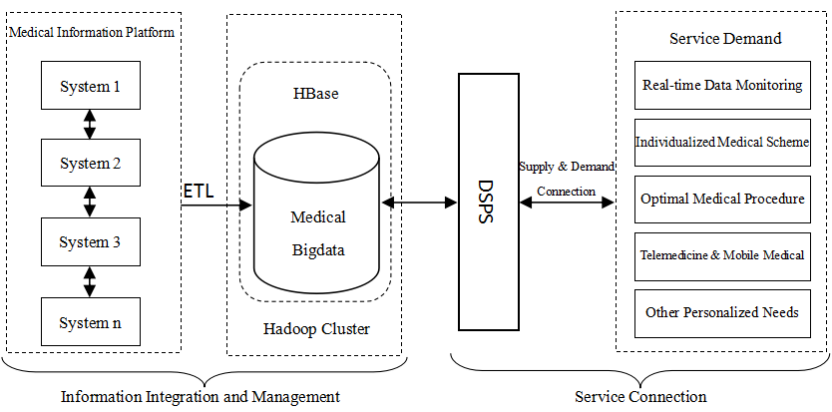
The data sharing and processing system architecture.

Integrated and shared medical data provide the technical support to realize these services. As previously stated, this innovative model could be achieved by using the DSPS (Figure 4). This system is based on mobile Internet technology that is driven by the big data value chain, and it connects the patients with different medical service providers, including doctors at the hospitals, family doctors, pharmaceutical companies, and other relevant participants in the medical system. The advantage of this connection is making information sharing possible by breaking the constraints of time and space.

First, based on Hadoop software, it would be practical to construct the hierarchical medical data-sharing platform that can realize medical big data integration and sharing. The integration scheme based on Hadoop platform consists of three parts. The first part consists of the medical institutions and the clinical information systems (EMR, PACS, LIS, etc), and the message engine would be based on Apache Camel. The second part utilizes the enterprise information portal (EIP) to design the routing rules and process flow. The third part is the Hadoop platform that is composed of the Hadoop cluster, Zookeeper, and HBase cluster.

Second, it is possible to explore the evolution of structures, agent behavior, and interactive form of subsystems in this model by using the system dynamics methods.

Third, it is feasible to develop the mobile application system, which includes the modules of authentication, appointment registration, electronic medical records, prescriptions, and test results query.

These 3 steps provide the necessary theoretical basis, technical support, and ways to develop this innovative model.

This huge data platform will take a single sign-on mode. Users need to log in, and based on the confidentiality level of the dataset, the users will have different levels of authority for access. Single sign-on unified identity authentication and authority control technology and strict control of user access are some of the ways to effectively ensure the safety of such large data applications. The system is based on the Hadoop platform. The cluster consists of a master node and a slave node. The master node needs to install and configure Hadoop, Hive, Sqoop, and MySQL in order to manage the cluster. Slave node only needs to install Hadoop; it can also be configured similar to the master node.

To ensure data privacy, the protection model mainly includes two aspects. One is the user-querying privacy protection; the query content cannot be leaked out. The second is data privacy. This protection model is based on the key. The data is protected by the form of anonymous processing based on the third party and query split. In this way, the data platform cannot connect the privacy content query with the users, thus protecting the user-querying privacy.

The application IoT technology in medicine covers almost all aspects, including medical practice, remote monitoring and home care, medical information, hospital first aid, medical equipment and medical waste monitoring, blood bank management, and infection control. An important application of IoT in medical information technology is mobile medical services, which are based on wireless LAN (local-area network) technology and RFID (radio-frequency identification).

In the DSPS, the Internet of Things (IoT) technology can be used to materialize the intelligent and real-time management of patients and the related systems such as digital collection, processing, storage, transmission, and sharing of information. The intelligent character recognition (ICR) technology is used at the hospitals to build the main index of patients and drugs. Using bar code scanning and RFID technology, hospitals have an accurate information confirmation and recognition system. This technology allows doctors to receive abundant data using the mobile sensor device and the medical instrument, which are both suitable for household use. IoT technology can support the collection of all kinds of vital signs data whenever and wherever possible. It can then automatically transfer the data to hospitals. Data mining and machine learning can be used to discover the hidden knowledge from these data. Interconnection technology can integrate patients and processes to allow for the standardized management of processes.

The main data collected by using IoT may include basic patient information (eg, full name, gender, date of birth, phone number, social security card number or ID number, and photo), medical information (eg, blood type, disease status such as diabetes, epilepsy, and hypertension), patient registration and treatment number, and medical treatment information (ie, medical records). It can also include the doctor’s electronic clinical orders (combined with mobile operation), patient medication treatment records (combined with medicare electronic settlement and payment), and patient tracking and positioning. Medical staff can access all these medical records both via portable hand held devices and desktop computers.

Telemedicine services can alleviate the queuing problem and reduce the cost of transportation for patients. It allows health care professionals to efficiently monitor patients’ indicators and give suggestions at any time. It is a low cost, rapid, and stable health monitoring method that can also extend medical services to subhealthy people, elderly patients, and patients with chronic diseases.

### Perspective

Surely, we need more research and dialogue on these issues and the impact associated with this innovative model of hierarchical medical system based on big data and mobile Internet. This model is likely to provide a new perspective and strategic choice of health care service, not only for China, but also for other countries.

## Abbreviations

DSPS: data sharing and processing system
EIP: enterprise information portal
EMR: electronic medical record
ICR: intelligent character recognition
IoT: Internet of Things
LAN: local-area network
LIS: laboratory information system
PACS: picture archiving and communication system
RFID: radio-frequency identification
RMS: regional medical consortium
